# Gene expression dynamics of natural assemblages of heterotrophic flagellates during bacterivory

**DOI:** 10.1186/s40168-023-01571-5

**Published:** 2023-06-15

**Authors:** Aleix Obiol, David López-Escardó, Eric D. Salomaki, Monika M. Wiśniewska, Irene Forn, Elisabet Sà, Dolors Vaqué, Martin Kolísko, Ramon Massana

**Affiliations:** 1 Department of Marine Biology and Oceanography, Institut de Ciències del Mar (ICM-CSIC), Passeig Marítim de la Barceloneta 37-49, Barcelona, Catalonia 08003 Spain; 2 Institute of Parasitology, Biology Centre, Czech Academy of Sciences, České Budějovice, Czech Republic; 3 Faculty of Science, University of South Bohemia, České Budějovice, Czech Republic

**Keywords:** Bacterivory, Heterotrophic flagellates, Metatranscriptomics, Functional genes, Unamended incubations, Phagocytosis, Peptidases, Glycosidases

## Abstract

**Background:**

Marine heterotrophic flagellates (HF) are dominant bacterivores in the ocean, where they represent the trophic link between bacteria and higher trophic levels and participate in the recycling of inorganic nutrients for regenerated primary production. Studying their activity and function in the ecosystem is challenging since most of the HFs in the ocean are still uncultured. In the present work, we investigated gene expression of natural HF communities during bacterivory in four unamended seawater incubations.

**Results:**

The most abundant species growing in our incubations belonged to the taxonomic groups MAST-4, MAST-7, Chrysophyceae, and Telonemia. Gene expression dynamics were similar between incubations and could be divided into three states based on microbial counts, each state displaying distinct expression patterns. The analysis of samples where HF growth was highest revealed some highly expressed genes that could be related to bacterivory. Using available genomic and transcriptomic references, we identified 25 species growing in our incubations and used those to compare the expression levels of these specific genes.

Video Abstract

**Conclusions:**

Our results indicate that several peptidases, together with some glycoside hydrolases and glycosyltransferases, are more expressed in phagotrophic than in phototrophic species, and thus could be used to infer the process of bacterivory in natural assemblages.

**Supplementary Information:**

The online version contains supplementary material available at 10.1186/s40168-023-01571-5.

## Background

Understanding the activity and functions of microbial communities in the ocean is fundamental to predict how marine ecosystems will be affected in the context of global change [1]. Marine microbes, both prokaryotes and microbial eukaryotes (protists), form the base of marine food webs and alterations in their composition and activities could directly impact biogeochemical cycles at a global scale [2]. Currently, increases in surface seawater temperature are amplifying the stratification of the water column, thus hampering mixing and the delivery of nutrients from the deep ocean to upper layers [3]. These changes are predicted to promote smaller microorganisms in the ocean [3, 4] and uncouple bacterial production from grazing mortality [5]. Consequently, bacterial biomass could increase, thus producing an imbalance between carbon recycling and carbon export to the deep ocean. Even though bacterivory is central in marine food webs, both the players and the genes they use are still largely uncharacterized.

The use of multi-omics techniques has completely changed the field of microbial ecology, providing new approaches to study microbial diversity and functions. Given the complexity of eukaryotic genomes and the fact that the presence of an eukaryotic gene informs little about its in situ function, metatranscriptomics has been the preferred approach to study the activity of microbial eukaryotes [6, 7]. These studies can benefit substantially from the generation of reference genomes of dominant marine species, and new genomes from uncultured protists have been recently obtained by metagenomics [8–10] and single-cell genomics [11–14]. Recent studies using metatranscriptomics on protist communities have broadened our knowledge on different topics [15] such as trophic strategies [16], diel and seasonal cycles [17, 18], nutrient responses [19, 20], or functional biogeography [21]. The application of these tools to study bacterivory in the ocean is becoming very promising.

Heterotrophic flagellates (HF) are main bacterial grazers in the ocean [22], and at the same time, the most understudied component of the marine microbiome [23]. These microscopic unpigmented protists (2–5 µm), found in the photic ocean at concentrations up to 10^3^ cells mL^−1^ [24], play a crucial role in the microbial loop by channeling carbon to higher trophic levels, remineralizing inorganic nutrients, and keeping bacterial abundances in balance [25, 26]. HFs predate on bacteria through phagocytosis, the uptake of the prey through membrane invagination and its digestion in an acidic environment [27]. Phagocytosis is an ancient evolutionarily conserved process with strong implications in the origin and evolution of eukaryotes [28, 29]. It has been deeply studied in metazoan immunity cells, given its role as a defense mechanism in human immunity [30], but only few studies have investigated the genes for phagocytosis as a nutritional process [12, 31–33]. In one of these, a machine-learning approach using reference genomes was performed to predict a phagotrophic trophic style [33].

Nevertheless, it is yet unclear which functional genes involved in phagocytosis are being expressed by marine HF species during bacterivory, mainly due to the lack of model organisms and the high phylogenetic diversity of HF assemblages. Some recent studies have explored this question by differential gene expression in cultured strains [34, 35]. However, as many of the dominant HF species in the ocean are not available in culture [36], we are still missing a large fraction of the molecular processes involved in marine bacterivory. Unamended incubations have proven to be a good approach to promote a pulse of bacterivory from natural HF assemblages [37], which facilitates a subsequent study of the gene expression of the stimulated uncultured species. We recently used this approach to follow the functional dynamics of a few uncultured MAST species by combining metatranscriptomics and single-cell amplified genomes [38]. Thus, the combination of unamended incubations and metatranscriptomics could allow identifying highly expressed genes by bacterivorous co-existing species.

In the present work, we performed four unamended dark incubations of surface seawater collected in the Blanes Bay Microbial Observatory (BBMO) at different seasons of the year to explore the functional dynamics of the HF community < 3 µm during bacterivorous growth. Our specific objectives were (i) to analyze whether HF communities showed similar expression patterns in different incubations, (ii) to identify the genes that are highly expressed during the process of bacterivory, and (iii) to validate if these could be used to infer bacterivory in natural assemblages. Altogether, our study advances the understanding on the biological processes shaping HF communities and shines a light on the characterization of bacterivory in the ocean.

## Methods

### Sampling at the BBMO and experimental setup

We performed four incubations: July 2017 (‘Jul17’), March 2018 (‘Mar18’), November 2018 (‘Nov18’), and September 2020 (‘Sep20’). For each incubation, surface seawater (see Fig. S1 for a schematic overview) was collected and pre-filtered through a 200-µm nylon mesh at the BBMO, a well-studied coastal sampling station located in the NW Mediterranean Sea [39]. In situ temperature was measured using a CTD probe. Carboys containing the seawater were covered with opaque plastic bags to avoid light penetration and transported to the laboratory in less than 2 h. There, 50 L of seawater was gravity-filtered through 3-µm pore-size polycarbonate filters into a polycarbonate carboy (Nalgene) and incubated in the dark for 5–10 days at in situ seawater temperature. The carboy was gently rolled on the floor once every 24 h to promote water mixing. Once a day, we sampled 2 L of seawater for RNA sequencing and filtered them through 0.6-µm pore-size (47/142 mm ø) polycarbonate filters using a peristaltic pump (~ 10-min filtration time). Samples were stored at − 80ºC after filtration. We also sampled 5 mL of seawater every 12–24 h and fixed them with glutaraldehyde (1% final concentration) for microbial counts. We stained the fixed samples with 4’,6-diamidino-2-phenylindole (DAPI) and filtered through 0.2-µm pore-size (25 mm ø) black polycarbonate filters. We manually counted cell abundances by epifluorescence microscopy of bacteria and heterotrophic flagellates (under UV light), phototrophic flagellates (UV and blue lights), and *Synechococcus* (blue and green lights).

### RNA extraction, sequencing, and read analyses

We performed RNA extraction and library preparation for Illumina sequencing as detailed in [38]. Briefly, we cut and vortexed the frozen filters in tubes containing Power Soil beads (Mobio) and extracted RNA using RNeasy Mini Kit (Qiagen) followed by a DNAse treatment with Turbo DNA-free kit (Ambion). Extracts were kept at − 80ºC until processing. We selected 21 samples for sequencing according to their RNA extraction yields, determined using Qubit RNA HS Assay Kit (Thermo Fisher Scientific). Polyadenylated transcripts were reverse transcribed to cDNA and enriched by 15 polymerase chain reaction (PCR) cycles at CNAG (https://cnag.cat/). RNASeq libraries were prepared with KAPA-Stranded mRNA-Seq Illumina (Roche-KAPA Biosystems). Sequencing was carried out in Illumina platforms HiSeq2500 for “Jul17” incubation, HiSeq4000 for “Mar18” and “Nov18” incubations, and NovaSeq6000 for “Sep20” incubation. Paired-end reads (2 × 100 bp) were obtained with a sequencing depth of 15 Gbp, except for “Sep20” incubation, with 25 Gbp.

We trimmed metatranscriptomic Illumina reads for adapters and filtered them for phred scores of ≥ 20 and length ≥ 75 bp with Trimmomatic v0.38 [40] (Fig. S2). In order to characterize the taxonomic dynamics of the incubations, we followed the pipeline described in [41] to extract and classify 18S rRNA fragments from the obtained Illumina reads, using version 5 of eukaryotesV4 database (https://github.com/aleixop/eukaryotesV4).

### Assembly, annotation, and quantification of transcripts

We first identified and removed ribosomal RNA fragments from quality-filtered reads using SortMeRNA v3.0.3 [42] with default parameters. We then co-assembled the remaining reads using rnaSPAdes v3.14.1 [43] with default parameters and obtained a single assembled metatranscriptome per incubation (Fig. S2). For each one, we removed sequences shorter than 200 bp with VSEARCH v2.17.0 [44] and kept the longest isoform of each gene. We taxonomically classified the transcripts using Kaiju v1.8.2 [45] in MEM mode with the nr + euk database and parameters ‘-x -m 11’ as in [46] and removed transcripts associated to prokaryotic taxa and viruses. We translated the transcripts to proteins using GeneMarkS-T v5.1 [47] with minimum length of 200 bp and default parameters and removed transcripts that could not be translated to a protein. We quantified the expression of the preliminary obtained transcripts per incubation in each sample using Salmon v1.8.0 [48] in mapping-based mode. We considered a gene as expressed if it had ≥ 2 transcripts per million (TPM) in at least one sample [49] and removed the transcripts below this threshold. With these final metatranscriptomes, we did a second quantification with Salmon and obtained the expression profiles for each sample. We functionally annotated the predicted proteins using eggNOG-mapper v2.1.2 [50, 51]. We performed most functional analyses using KEGG ortholog (KO) annotations [52] and its higher-order associated classifications (BRITE and Pathway). To do so, we generated an expression profile KO table by pooling the TPM values from transcripts associated to each individual KO, each related to a distinct functional gene with all its intraspecific and interspecific variants.

### Detection in the incubations of species with known genomic data

We built a reference protein database of 1038 eukaryotic genomes and transcriptomes by combining EukProt database version 3 [53] and 50 single amplified genomes prepared from BBMO samples. In order to detect the presence of these species in our incubations, we aligned the metatranscriptomes to this protein database using DIAMOND blastp v2.0.14 [54] in “sensitive” mode. We kept the top scoring hits for each transcript after selecting alignments with > 90% identity and a minimum of 50 amino acids. We removed transcripts having more than one top hit (same *e* value) with different reference genomes (1.6% of the cases). With this, we identified 51 species that had a reasonable similarity in the incubations and at least 100 transcripts in one incubation. Then, we verified if the signal detected derived from the same exact species (or a closely related one) by mapping the retrieved transcripts to the coding sequences (CDS) of the reference species at the nucleotide level using blastn v2.7.1 [55]. We considered a species present in the incubations when the transcripts and the reference CDS had a median identity > 99%. We selected 25 species, annotated their reference genomes/transcriptomes using eggNOG-mapper and quantified their gene expression by mapping the unassembled metatranscriptomic reads to their CDS sequences using Salmon. We normalized the obtained read counts using the effective length of each mapped CDS sequence (i.e., we divided mapped read counts by the effective length reported by Salmon) and converted them to integer counts ranging from 0 to 10^6^ using a pseudo-count as in Salazar et. al [56]. Then, we corrected the obtained expression profiles using TMM transformation and converted them to pseudocounts per million using edgeR [57]. We assigned trophic mode to selected species according to common assumptions in the literature. Thus, we considered Diatomeae and all Archaeplastida species to be phototrophs, except for Picozoa (heterotrophs) [14]. In groups of pigmented microorganisms known to harbor multiple mixotrophic species (Prymnesiophyceae, Bolidophyceae, and Dictyochophyceae), we considered all species to be mixotrophs [58–60]. We labeled as heterotrophs the rest of groups, as they encompass unpigmented species. For taxa with no detailed species assignment, we used the trophic mode of their closest cultured match in GenBank. ChrysophyceaeNA-sp1 was closest to *Paraphysomonas imperforata* at 99.1% identity (heterotroph), while Micromonas-sp1 was closest to *Micromonas bravo* at 99.8% identity (phototroph).

### Statistical analyses

We performed all general analyses using R v4.1.1 [61] with packages tidyverse v1.3.1 [62] and vegan v2.5.7 [63]. We divided the samples of each incubation into “lag”, “growth”, and “decline” states according to their position in the HF growth curve assessed by microscopy. We validated this classification using plotPCA function in package DESeq2 v1.32.0 [64] using transcript counts obtained by Salmon followed by a variance stabilizing transformation (Fig. S3). From the list of 359 highly expressed genes (i.e., KO entries) at the growth state in the incubations (Table S1), we identified 104 housekeeping genes, later used for normalizing gene expression functions, by choosing ribosomal proteins and other genes generally used in the literature [65, 66].

## Results

### Growth and taxonomic dynamics

**Fig. 1:**
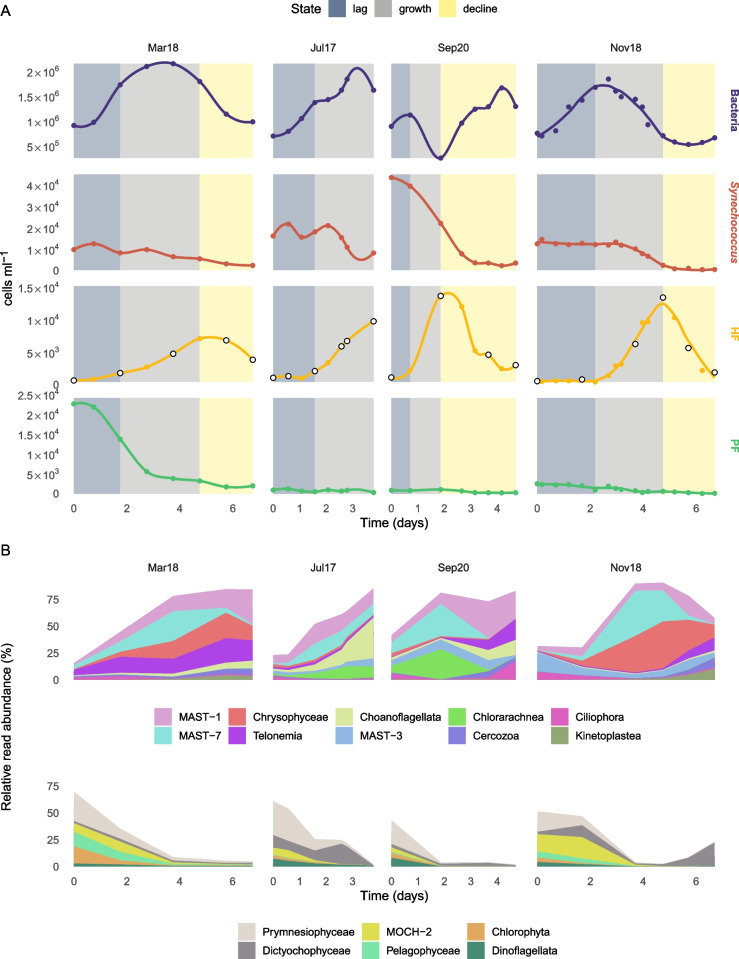
Cell counts and taxonomic dynamics during the four incubations. **A** Cell counts of heterotrophic bacteria, *Synechococcus*, heterotrophic flagellates (HF), and phototrophic flagellates (PF) conducted by epifluorescence microscopy. The background of the plots is colored by the different incubation states of the HF community (“lag”, “growth”, and “decline”). White dots in HF curves represent time points from which we obtained metatranscriptomic data. **B** Relative read abundance of the main taxonomic eukaryotic groups during the incubations as seen by 18S-V4 mTags. Groups are divided into 2 plots by their overall dynamics: increasing (upper panels) or decreasing (bottom panels) their relative abundance

We conducted a total of four unamended incubations between July 2017 and September 2020 following similar experimental procedures (Table 1; Fig. S1). For each one, we followed the growth dynamics of bacteria and picoeukaryotes by epifluorescence microscopy. In all cases, there was an initial peak of bacteria followed by a peak of heterotrophic flagellates (HF) and a continuous decrease of photosynthetic populations (Fig. 1A). Initial bacterial abundances were around 7–9 × 10^5^ cells ml^−1^ and peaked to ~ 2 × 10^6^ cells ml^−1^ at 4–5 days in the first three incubations, while in “Sep20” the bacterial peak was earlier and lower. The initial peak of bacteria was followed by a peak of HF, which increased from 5–10 × 10^2^ cells ml^−1^ to 7–13 × 10^3^ cells ml^−1^. In the absence of light, the phototrophic flagellates (PF) presented a steady decrease from the initial counts of 1–3 × 10^3^ cells ml^−1^, with the exception of “Mar18” incubation, where initial cell abundances were one order magnitude higher due to a bloom of very small cells (1–2 µm). *Synechococcus* cell abundances steadily decreased from initial abundances about 2 × 10^4^ cells ml^−1^ in the first three incubations and twice these initial values (and a more marked decrease) in “Sep20”. Following the HF dynamics in Fig. 1A, we classified the incubations into three states (“lag”, “growth”, and “decline”).

**Table 1:** Overview of the incubations

Incubation	Sampling date	Water temp (ºC)	metaT samples	Num. transcripts (10^3^)	Total size (Mbp)	N50 (bp)	KEGG annotated (%)
Mar18	06/03/2018	14	5	294	187	1112	31.4
Jul17	04/07/2017	24	6	225	162	1438	32.9
Sep20	15/09/2020	24	4	410	201	799	22.4
Nov18	05/11/2018	19	6	350	228	1216	30.6

General information of the incubations and statistics of the final metatranscriptomes

Taxonomic groups with chloroplast-harboring species—Prymnesiophyceae, Dictyochophyceae, MOCH-2, Pelagophyceae, Chlorophyta, and Dinoflagellata—showed a clear decreasing trend along the incubations (bottom panels in Fig. 1B). These accounted for 43–70% of relative read abundance at the beginning of the incubations and were nearly absent towards the end. Dictyochophyceae in “Nov18” was the only exception to this trend, as its relative abundance increased from 1% at around day 4 to 22% at the final time. Heterotrophic protists (upper panels in Fig. 1B) initially represented 17–42% of the relative read abundance and explained most of the read signal during the “growth” state (83–91% total relative read abundance). The most abundant groups considering the 4 incubations were MAST-1 and MAST-7. Chrysophyceae and Telonemia were also very abundant in “Mar18” and “Nov18” incubations (the latter also in “Sep20”). In terms of the overall development of main taxonomic groups, incubations grouped by pairs: “Jul17” and “Sep20” were closer, as well as “Mar18” and “Nov18”.

### General functional dynamics

We generated four de novo metatranscriptomes by coassembling reads and curating the transcripts sets (see Fig. S2 for a schematic overview of the process). The final datasets contained 2–4 × 10^5^ transcripts with a N50 ranging from 799 to 1438 bp (Table 1). Using KEGG database, we could functionally annotate approximately a third of the transcripts (Table 1), which represented around half of total TPM in each incubation (Fig. S4). A PCA plot with the normalized counts for these transcripts roughly validated the three states considered here (Fig. S3).

**Fig. 2:**
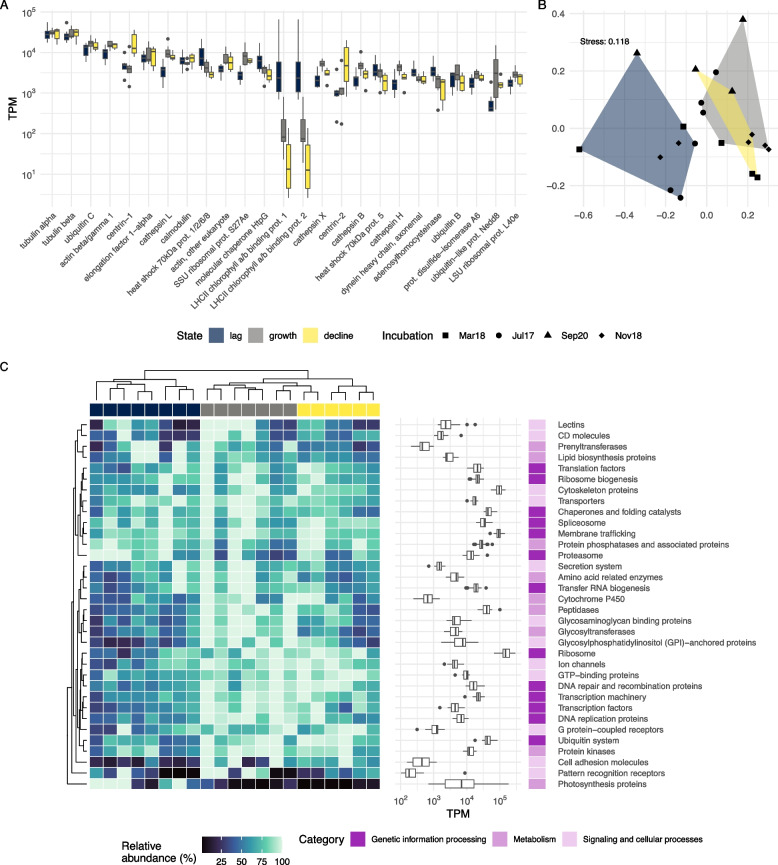
General functional dynamics of the four incubations. **A** Expression values per sample of the 25 most expressed KOs (KEGG orthologs) in the incubations represented by boxplots colored by incubation state. **B** Non-metric multidimensional scaling (NMDS) plot using Bray–Curtis dissimilarities between the expression of KOs in the different samples. Samples are grouped by their incubation state. **C** Heatmap displaying the expression of main categories as represented by KEGG BRITE classifications in all samples (i.e., each column represents a sample). Values shown are computed by scaling TPM values to a 0–100 scale per category and incubation (i.e., TPM values belonging to a category and incubation are divided by their maximum value). Boxplots display the actual TPM values per sample of each category

The most expressed KEGG orthologs (KOs) considering all incubations (Fig. 2A) were associated to proteins involved in cytoskeleton structure, such as actin, tubulin, and centrin. These showed relatively similar levels of expression along incubation states except for centrins, which displayed a higher expression during “decline” state. Ribosomal proteins and elongation factors, involved in protein synthesis, were also highly expressed, with rather constant expression levels along time. Other highly expressed KOs were calmodulin and ubiquitin, proteins related to signal transduction, which followed the same pattern; and cathepsins L and X, cysteine peptidases that were more expressed in the “growth” state. Among the highly expressed KOs, there were also 2 photosynthesis-related proteins (chlorophyll a/b binding proteins) that exhibited high TPM numbers in “lag” state and a dramatic decrease at the “growth” and “decline” states. The incubations exhibited similar overall dynamics when comparing their gene level (KO) TPMs in a NMDS plot (Fig. 2B). Thus, samples seemed to be organized by time of the incubation rather than by incubation. In terms of states of the incubation, “lag” was clearly differentiated from the rest, while “growth” and “decline” states displayed a clear overlap.

Higher-level KO annotation, as represented by KEGG BRITE categories, showed similar structuring between states with “growth” and “decline” samples clustering together and “lag” samples forming a separate cluster (Fig. 2C). The decrease along incubation time in the expression of photosynthesis-related proteins was very apparent (Fig. 2C), with virtually no expression in “decline” samples. Apart from this clear pattern, the dynamics of the remaining categories were less marked and could be roughly divided into 3 different trends: (i) functions with genes exhibiting high expression through all incubation states, (ii) those showing higher expression in both “growth” and “decline” states, and (iii) those more expressed in the “growth” state. The first group included constitutive processes of the cell, such as spliceosome, translation factors, or chaperones, as well as membrane trafficking processes and transporters. In the second category, ribosome was the most expressed category, followed by ubiquitin, GTP-binding and transcription- and replication-related proteins. The third group contained peptidases, GPI-anchored proteins, and glycosyltransferases.

### Most expressed genes during “growth” state

**Fig. 3:**
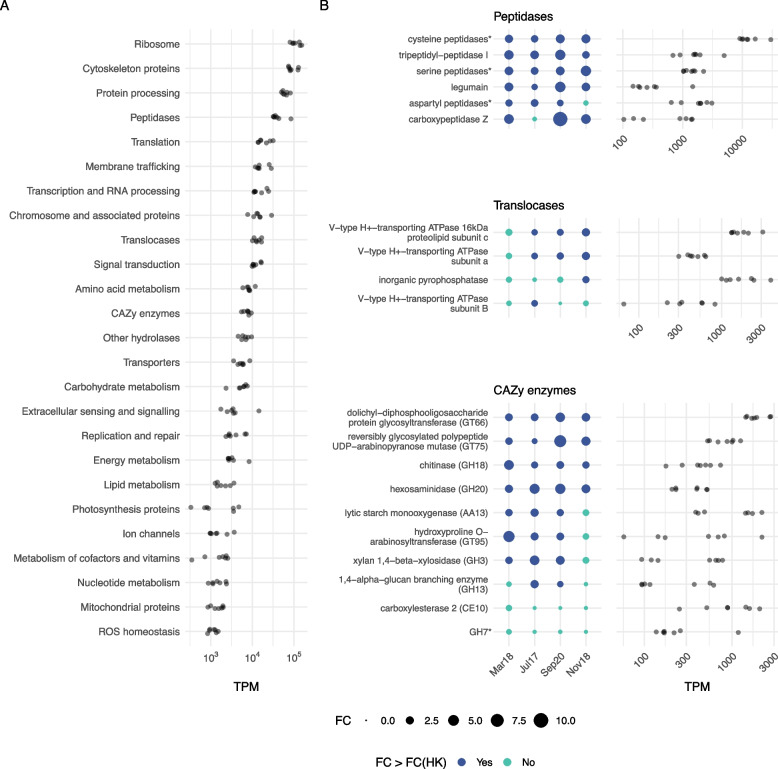
Most expressed functions and genes in the growth state of the incubations. **A** Expression values (TPM) of the 359 most expressed genes in “growth” samples pooled into custom categories, delineated to report each KO to a single category (see Table S1 for further details). **B** Fold change (FC) between “lag” and “growth” incubation states and TPM expression values of genes (KOs) annotated as peptidases, translocases (proton pumps), and CAZy enzymes. Dots representing FC larger than the average FC of housekeeping genes are colored in dark blue. KOs displaying overlap in functional annotations (i.e., different KOs associated to the same transcript) needed to be grouped into broader sets (asterisk, see Fig. S5 for further details). “Cysteine peptidases” include cathepsins B, F, H, K, L, O, and X, as well as KDEL-tailed endopeptidase and xylem cysteine peptidase; “aspartyl peptidase” includes cathepsins D and E, phytepsin and saccharopepsin; “serine peptidase” includes cathepsin A, serine carboxypeptidase-like clades I and II and vitellogenic carboxypeptidase-like protein. For CAZy enzymes, “GH7” groups cellulose 1,4-beta-cellobiosidase and cellulase

Based on the dynamics of bacteria and HF in all incubations, it could be deduced that the “growth” state was the period when the bacterivory process had the highest relative importance. So, we focused on the most expressed genes (KOs) during that state. We obtained a list of 359 highly expressed KOs (those with > 500 TPM on average in one of the incubations), which we then grouped into a custom system of 24 categories partially based on KEGG BRITE and Pathway hierarchies, assigning each KO to a single category (Table S1) to avoid duplicated signal. These general functions presented comparable gene expression levels when putting together all “growth” samples from the four incubations (Fig. 3A). Some of the most expressed categories were related to processes generally considered as constitutive or housekeeping, as already seen in general dynamics (Fig. 2): ribosomal and cytoskeleton proteins, protein processing, translation, replication, or transcription. However, other categories related to metabolism also emerged, such as peptidases, CAZy enzymes, or other hydrolases, as well as translocases (mainly including proton pumps) and membrane trafficking proteins (Fig. 3A).

Following the data revealed in Fig. 3A and past evidence of their role in phagocytosis (see the “Discussion” section for further details), we analyzed in detail the KOs of three promising functional categories, namely peptidases, translocases (proton pumps), and CAZy enzymes (Fig. 3B). We first assessed the overlap in the functional annotation of transcripts to avoid having different KOs with the same transcript associated. This was particularly critical within peptidases, in which the 20 KOs were grouped in 6 unique categories (Fig. S5). We then computed the average fold change (FC) of the 104 housekeeping genes (HK; Table S1) between “lag” and “growth” states per incubation and used this value as baseline to compare the expression FC of the rest of the genes. All peptidase genes had a higher expression in the “growth” state than HK genes in at least 3 of the 4 incubations (Fig. 3B), suggesting an upregulation during growth by bacterivory, with an average FC > 3 (i.e., their expression increased more than 3 times from “lag” to “growth” states). Among these, cysteine peptidases (including 7 cathepsin types, see Fig. S5) were by far the most expressed in “growth” state (Fig. 3B). For translocases, genes related to V-type ATPase displayed different patterns, with two subunits (a and c) being more expressed than HK genes in 3 incubations and subunit B in only one. We also detected this latter trend with inorganic pyrophosphatase, which was one of the most expressed genes related to proton pumps in the incubations (Fig. 3B). For CAZy enzymes, two glycoside hydrolases (GH18 and GH20) and two glycosyltransferases (GT66 and GT75) were more expressed than HK genes in all the incubations. Carboxylesterase 2 and GH7 genes showed the opposite trend, with a lower FC than HK in all cases. GT66, AA13, and CE10 were the most expressed CAZy genes in “growth” state (Fig. 3B).

### Gene expression at the species level

**Fig. 4:**
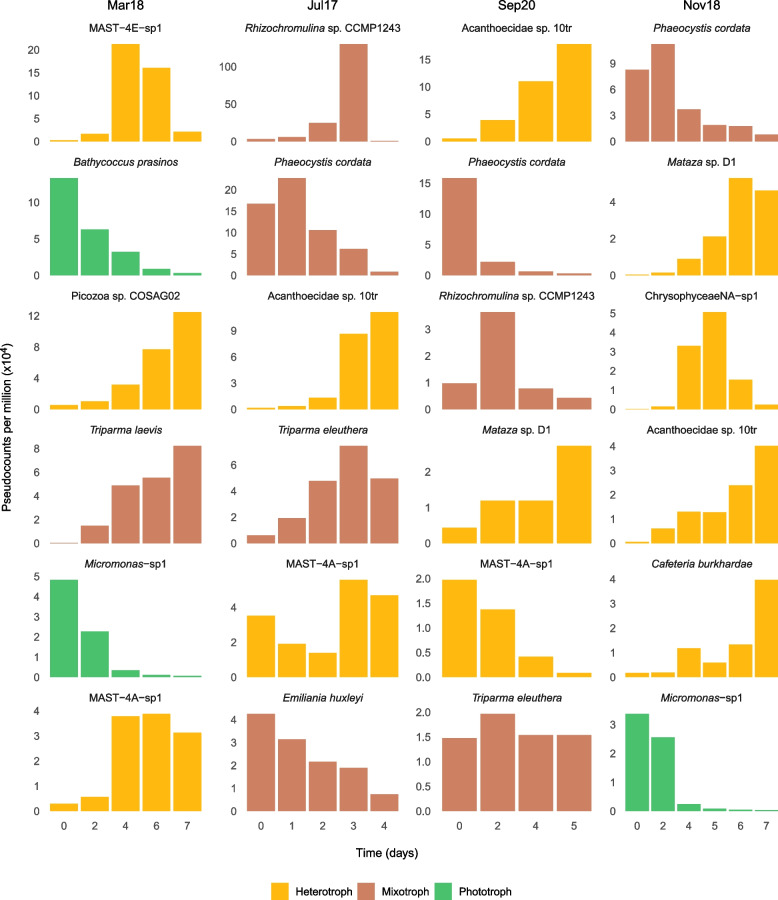
Expression dynamics of a selection of species with genomic data found in the metatranscriptomes. See the full list of the 25 detected species in Table 2 and the display of their expression dynamics in Fig. S7. Values represent pseudocounts per million, obtained after correcting the abundance profiles by gene length and sequencing depth (see the “Methods” section for details)

After looking at the expression dynamics at the community level, we investigated which species with genomic/transcriptomic data available were present in the incubations, which would allow a detailed analysis of their gene expression in our metatranscriptomes. By mapping the transcripts to a custom protein database of eukaryotic species (see the “Methods” section for details), we obtained a preliminary list of 51 candidate taxa (Fig. S6). The comparison between the species coding sequences and their associated transcripts revealed cases of virtually identical sequences (green bars in Fig. S6) but also cases of transcripts having median identity ranging from 90 to 95% (red bars in Fig. S6) that could not be considered to derive from that species but from a highly related one. After filtering these cases, we obtained a final list of 25 species present in our incubations (Table 2). In terms of taxonomic diversity, the list contained 12 Stramenopiles (several MASTs, Ochrophyta, and Bicosoecida), 6 Archaeplastida (Chlorophyta and Picozoa), 5 Haptista (Prymnesiophyceae), 1 cercozoan, and 1 choanoflagellate (Table 2). In general, heterotrophic species tended to increase their expression towards the middle and end of the incubation, while phototrophic species were highly expressed at the beginning and decreased along incubation time (Fig. 4). In the case of mixotrophic species, a mix of the abovementioned trends was seen, as well as some species displaying a rather steady expression (Fig. S7).

**Table 2:** Species with genomic data well represented in the metatranscriptomes

Species	Supergroup	Group	Source	Trophic mode	BUSCO (%)	Incubations present	Max reads recovered per sample (%)
*Bathycoccus prasinos*	Archaeplastida	Mamiellophyceae	genome	Phototroph	75.3	2	2.74
Micromonas-sp1*	Archaeplastida	Mamiellophyceae	single-cell genome	Phototroph	27.1	2	0.69
*Ostreococcus lucimarinus*	Archaeplastida	Mamiellophyceae	genome	Phototroph	78.1	1	0.62
Picozoa sp. COSAG01	Archaeplastida	Picozoa	single-cell genome	Heterotroph	21.6	1	0.02
Picozoa sp. COSAG02	Archaeplastida	Picozoa	single-cell genome	Heterotroph	32.1	1	0.03
*Pycnococcus provasolii*	Archaeplastida	Pycnococcaceae	transcriptome	Phototroph	55.3	1	0.01
*Chrysochromulina rotalis*	Haptista	Prymnesiophyceae	transcriptome	Mixotroph	53.7	1	0.11
*Dicrateria rotunda*	Haptista	Prymnesiophyceae	transcriptome	Mixotroph	36.1	1	0.28
*Emiliania huxleyi*	Haptista	Prymnesiophyceae	genome	Mixotroph	56.1	3	0.10
Isochrysidales sp. CCMP1244	Haptista	Prymnesiophyceae	transcriptome	Mixotroph	57.2	3	0.07
*Phaeocystis cordata*	Haptista	Prymnesiophyceae	transcriptome	Mixotroph	53.4	3	1.00
Acanthoecidae sp. 10tr	Opisthokonta	Choanoflagellata	transcriptome	Heterotroph	78.8	3	0.51
*Mataza* sp. D1	Rhizaria	Cercozoa	transcriptome	Heterotroph	77.7	2	0.10
*Cafeteria burkhardae*	Stramenopiles	Bicosoecida	genome	Heterotroph	67.1	1	0.10
*Triparma eleuthera*	Stramenopiles	Bolidophyceae	transcriptome	Mixotroph	53	3	0.19
*Triparma laevis*	Stramenopiles	Bolidophyceae	transcriptome	Mixotroph	40.8	2	0.23
ChrysophyceaeNA-sp1*	Stramenopiles	Chrysophyceae	single-cell genome	Heterotroph	21.2	1	0.13
*Leptocylindrus hargravesii*	Stramenopiles	Diatomeae	transcriptome	Phototroph	65.1	1	0.05
*Rhizochromulina* sp. CCMP1243	Stramenopiles	Dictyochophyceae	transcriptome	Mixotroph	67.9	2	1.90
MAST-1C-sp1*	Stramenopiles	MAST-1	single-cell genome	Heterotroph	3.9	2	0.08
MAST-1D-sp2*	Stramenopiles	MAST-1	single-cell genome	Heterotroph	11.4	1	0.02
MAST-3C-sp2*	Stramenopiles	MAST-3	single-cell genome	Heterotroph	31.4	1	0.04
MAST-4A-sp1	Stramenopiles	MAST-4	single-cell genome	Heterotroph	73.8	4	0.28
MAST-4E-sp1	Stramenopiles	MAST-4	single-cell genome	Heterotroph	57.3	2	1.56
MAST-8B-sp1*	Stramenopiles	MAST-8	single-cell genome	Heterotroph	18.4	2	0.05

The 25 species are displayed with general taxonomy, genome completeness (BUSCO), and quantification information in the incubations. Species marked with an asterisk were retrieved using our own BBMO SAG collection, while the rest were retrieved using EukProt database

**Fig. 5:**
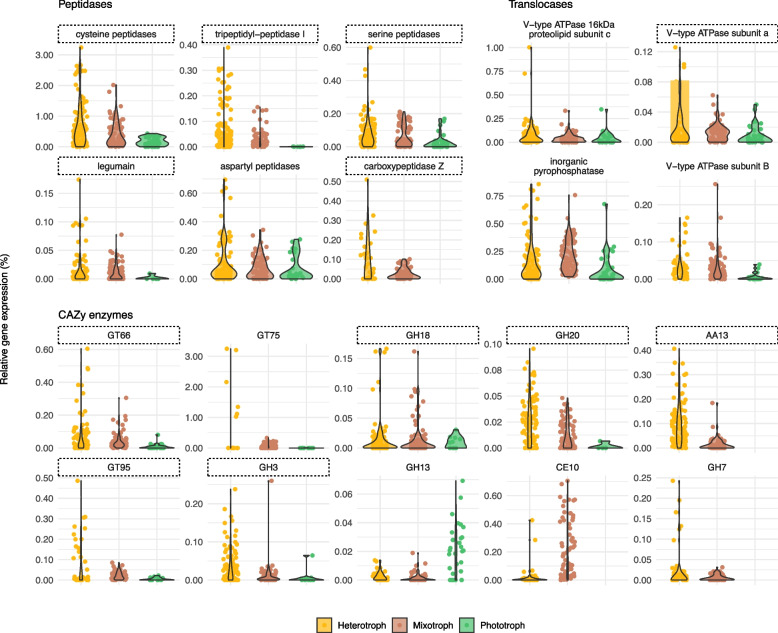
Expression of selected genes in species with different trophic modes. Points represent the relative expression of the gene in a single species and sample. Values were computed by dividing the expression of the selected gene by the total expression for each species and sample. Values are separated by the trophic mode of the species they come from (Table 2). Genes within dashed rectangles have a higher relative expression in phagotrophs than in phototrophs and could be good candidates to infer bacterivory in natural assemblages (see Table S2 for more details)

Taking advantage of the different trophic modes represented by the 25 species (12 heterotrophs, 8 mixotrophs, and 5 phototrophs), we analyzed whether the expression of the genes identified as putatively relevant for bacterivory varied between nutritional strategies. The majority of these genes displayed higher levels of expression in phagotrophs compared to phototrophs (Fig. 5; Table S2). Within peptidases, cysteine peptidases were the ones with the highest relative expression values, with approximately one order of magnitude higher than the rest (Fig. 5). Relative gene expression differed depending on trophic mode, with heterotrophic species always displaying slightly higher values (around 2% of gene expression) than mixotrophic species and markedly higher than phototrophic ones (< 0.5%). The remaining peptidase genes displayed lower expression levels but generally followed a similar trend. In translocases, inorganic pyrophosphatase had the highest levels of relative expression and, together with the V-ATPase subunit a, was more expressed in heterotrophs than in phototrophs (Fig. 5). CAZy enzymes showed different trends. GH13 seemed to be more expressed in phototrophs, CE10 in mixotrophs, and GT66, GH18, GH20, AA13, GT95, and GH3 in heterotrophs. As revealed by functional annotations, genes encoding enzymes CE10 and GH7 were not present in genomes/transcriptomes from the selected phototrophic species, and AA13 had a single sequence annotated in *Pycnococcus provasolii*. Therefore, these did not display any relative expression value for phototrophs (Fig. 5). We selected the best candidates as marker genes for bacterivory by combining the results of a randomization test with 10,000 permutations (*p* value < 0.05), and the results of fold change reported in the previous section (Fig. 5; Table S2).

## Discussion

Marine heterotrophic flagellates (HF) remain largely undersampled, and very few ecologically relevant species are available in culture [23]. As a consequence, key processes in global biogeochemical cycles, such as the grazing on marine bacteria by small predators, still need to be well characterized. In this study, we performed four unamended incubations of coastal seawater in the dark to promote the growth of natural HF assemblages and assess their gene expression during bacterivory.

### Circumventing the lack of cultured representative HF species

The use of unamended incubations allowed a more than tenfold increase in cell densities of natural HF assemblages. Even after performing a polyA selection towards eukaryotic messenger RNA, the obtained metatranscriptomes contained a remarkable ribosomal RNA signal (around 3% of reads on average matched the V4 region of the 18S rDNA) due to its huge abundance in the cell [67]. We took advantage of this to assess the general taxonomic dynamics in the incubations, an approach supported by a previous study where we reported that the relative abundance of this rRNA signal was well correlated with the FISH counts of the target cells [38]. The most represented taxa belonged to MAST clades, Chrysophyceae and Telonemia, groups that have been identified to be highly abundant and widespread in the surface ocean [36]. Despite the general dominance of these taxonomic groups, differences emerged between the individual species growing in the incubations, thus highlighting the seasonality of protist communities in BBMO [68] and the large diversity of natural assemblages.

Most of the groups present in the incubations are known to be poorly represented in public databases, so we opted for a dual strategy to analyze our dataset. First, we performed de novo assemblies to analyze the expression patterns of the whole community. We could functionally annotate half of the transcripts in the assemblies, while the other half remained completely unknown. This issue was also reported in a global eukaryotic metatranscriptomic survey [21] and agrees with the estimation that currently 40–60% of microbial predicted genes cannot be assigned to a known function [69]. Second, we used taxonomic binning, as carried out in other studies [19, 70], to answer specific questions on some bacterivory-related genes. Although less than 15% of metatranscriptomic reads mapped to our reference genome dataset with > 90% identity (Fig. S8), this approach revealed interesting expression insights in relevant species-specific functional genes.

The general functional dynamics of the 4 incubations showed seemingly similar patterns of gene expression that could reflect some kind of functional redundancy, by which different sets of co-existing taxa are performing the same function in the ecosystem [71]. The most visible trend in functional dynamics was the expression decrease of photosynthesis-related genes to virtually zero, in agreement with the observed decrease of phototrophic flagellates and the fact that incubations were performed in the dark. Despite representing an obvious outcome of the experimental setup, this transition from phototrophy to heterotrophy in all the incubations highlights the reliability of our data to analyze the expression of bacterivory-related genes.

### Highly expressed genes during bacterivory

Some of the most expressed categories in “growth” samples (where bacterivory should be highest) belonged to constitutive processes, with genes involved in several cellular functions (like actin or tubulin), and we did not analyze them any further. Instead, we focused on other sets of highly expressed genes such as peptidases, translocases, and CAZy enzymes, as these could be promising targets in the study of bacterivory.

The majority of the highly expressed peptidase genes belonged to cathepsins. According to the overlap displayed in functional annotations, these clustered into separate groups by their catalytic type (cysteine, serine, and aspartyl peptidases). An exhaustive analysis should be performed to identify the phylogenetic relationships within these groups, as most of them were described in humans or model organisms [72, 73] and little is known for uncultured protists species. Cathepsins are mainly found in the lysosome, where they act as digestive enzymes degrading proteins in acidic conditions [74]. Their high gene expression during bacterivory is consistent with the fact that more than 60% of bacterial dry weight is composed by proteins [75]. Some peptidases have been previously found in phagosomes, such as cathepsin L in metazoan macrophages [76] or cathepsin D in the amoeba *Dictyostelium discoideum* [77]. A recent paper working with the bicosoecid flagellate *Cafeteria burkhardae* detected an abundant cysteine peptidase gene being differentially expressed during bacterivory [34], and other studies have reported their presence in mixotrophic algae [78], mixotrophic dinoflagellates [79], and MAST groups [38].

Within translocases, V-type ATPase and inorganic pyrophosphatase genes were among those highly-expressed in “growth” samples. V-type ATPase proton pumps are involved in the acidification of the lysosome, among other organelles [80], and it has been hypothesized that pyrophosphatase could play a similar role in protists not belonging to Opisthokonta and Amoebozoa supergroups [12, 81], where this protein is absent. The gene for this proton pump was more expressed than the V-type ATPase in the abovementioned *C. burkhardae* study, thus supporting this view [34]. We did not find a clear pattern of upregulation of the pyrophosphatase proton pump between “growth” and “lag” states, whereas V-ATPases-related genes showed contrasting trends depending on the targeted subunits. These belonged to different domains of the proton pump [80] with distinct roles (ATP hidrolysis in subunit B and proton translocation in subunits a and c) that might explain the observed patterns.

Carbohydrate-active (CAZy) enzymes are related to carbohydrate metabolism [82], including glycoside hydrolases (GH) and glycosyltransferases (GT). GTs create glycosidic bonds with a variety of organic substrates [83], although some of the reactions they catalyze can be reversible [84]. Actually, some GTs have been reported to have distinct enzymatic activities (hydrolysis among them) with differing pH optima [85]. GT66 gene, involved in the *N*-glycosylation of proteins [86]—a highly conserved metabolic process obligatory for viability in eukaryotes [87]—as well as GT75 gene, which can be associated to cell wall metabolism [88], were upregulated in “growth” state. The upregulation of GT66 entails an increase in protein glycosylation by *N*-glycans, which, among various functions, are involved in the functionality of some lysosomal proteins [89]. The high expression of GT75 could be due to either a biosynthetic pathway or an undescribed degradative process. In the case of GHs, they are responsible for carbohydrate hydrolysis [83], and they could be involved in bacterivory as digestive enzymes. It was recently reported that GHs account on average for 3% of predicted genes in four MAST-4 species [13], which are among the most abundant HFs in the ocean [36]. A gene encoding a chitinase belonging to GH18 was the most expressed GH in “growth” samples. Chitin is mostly found in metazoans, fungi, and diatoms [90], and the presence of chitinase in picosized HFs indicates that these enzymes may have other physiological functions that are still unknown [91]. Other highly expressed GH genes in our incubations were hexosaminidase (GH20) and xylan 1,4 − beta − xylosidase (GH3). Hexosaminidases are abundant components found in phagosomes of the parasite *Entamoeba histolytica* [32] and several GH20 genes were found in the genomes of MAST-4 and MAST-3 species [92]. Xylan is an important component in plant cell walls [93], and the enzyme 1,4 − beta − xylosidase is relevant to the production of biofuels [94]. Xylan also occurs in some red and green algae [95] and the high expression of GH3 genes in the incubations could be due to microscopic algae being ingested in our incubations. A CAZy enzyme not belonging to GH or GT, the lytic starch monooxygenase (AA13), also displayed high levels of expression. This enzyme was recently discovered [96] and is related to the degradative activity of recalcitrant polysaccharides [97].

Only three of the highly expressed genes reported here—cellulase (GH7), 1,4 − beta − xylosidase (GH3) and tripeptidyl-peptidase I—were present in the set of 474 genes predicting phagotrophic trophic mode in a previous comparative genomics model [33]. Considering that this model was built using reference genomes, differences between the approaches were expected, and this highlights the importance of adding expression data in the search of marker genes for phagocytosis.

### Expression of bacterivory genes at the species level

From the subset of more than 1000 species having genomic/transcriptomic data, we found 25 of them in the incubations. Ten of them had a partial genome obtained through single-cell genomics (SCG), highlighting both the number of uncultured taxa growing in the incubations and the power of SCG to access the genomes of environmentally relevant taxa. As expected, all the 25 species identified had a picoplanktonic size (≤ 3 µm), with the exception of the diatom *Leptocylindrus hargravesii* (20–200 µm). Technically, this species should not be detected in our datasets, as we performed the incubations with seawater prefiltered by 3 µm, and a possible explanation could be that we were targeting gametes produced for sexual reproduction [98] that were passing through filters. We reported additional species in the database that retrieved transcripts from the incubations with a median identity at the nucleotide level of 90–98%, indicating the presence of a close relative of the reference species (Fig. S6). If we take as an example MAST-4A and MAST-4B species available in EukProt, which are very close phylogenetically (2 bp difference at the V4 rDNA amplicon), and compare their coding sequences, the median identity we obtain is around 85% (Fig. S9). Altogether, it could be inferred that 25 of the reference species, together with some closely related ones, were growing in the incubations.

With the expression data retrieved from the 25 species we could test whether selected peptidases, translocases, and CAZy genes represented a larger share of the species expression in bacterivorous species compared to phototrophic ones, and thus could be related to the process of bacterivory. Given that phototrophs may have been in suboptimal conditions under dark conditions, one could argue that this analysis could be biased towards heterotrophic modes. However, we did not detect strong differences in relative expression for phototrophic species between initial samples, where PF abundance was highest and the absence of light was just starting, and the rest of the incubations (Fig. S10). Transcriptomic studies in phototrophic species growing in optimal light conditions need to be done to further validate their low expression of bacterivory genes.

Our analysis revealed that several peptidase genes were generally more expressed in species having a phagotrophic mode of nutrition (heterotrophs and mixotrophs), with cysteine peptidases representing up to 3% of the total gene expression of some species. This suggests that, although these could participate in other cellular processes, they have a key role in bacterivory as digestive enzymes. Proton pumps (translocases) did not display these marked differences between trophic modes, except for one V-ATPase subunit. For CAZy enzymes, GT66, GH18, GH20, AA13, GT95, and GH3 were the genes displaying the most marked differences of relative expression between phagotrophic and phototrophic species. Thus, these could be key players in bacterivory, with a role in the digestion of ingested bacteria in the phagolysosome. Although the transcripts associated to the 25 identified species only represent 2.5% of the total signal in the 4 metatranscriptomes (data not shown), this analysis represents a proof-of-concept of what can be achieved with more reference genomes from species that are currently uncultured.

## Conclusions

Using a combination of incubations to promote the growth of natural HF assemblages and metatranscriptomic data, we obtained high-quality gene expression data related to bacterivory, a critical process in global biogeochemical cycles that still needs to be well characterized. Our results indicate that different HF communities follow similar functional profiles and that genes encoding peptidases and CAZy enzymes are highly expressed during the process of bacterivory. A more detailed analysis at the species level revealed that among others, cysteine peptidases, together with some glycoside hydrolases and glycosyltransferases, are key players in this process and could be used to infer bacterivory in natural assemblages. A further study of these genes and their expression in multiple and diverse taxa could reveal the intricate mechanisms of marine bacterivory, which in turn could improve predictions on the alteration of marine ecosystems due to global change.

### Supplementary Information

**Additional file 1: Fig. S1.** Overview of the experimental setup. **Fig. S2.** Overview of the bioinformatic processing of the metatranscriptomic reads. **Fig. S3.** PCA plots used for validation of the different incubation states based on normalized read counts per transcript. Each point represents a sample, and the value next to it represents the time of incubation expressed as days. **Fig. S4.** (A) Percentage of transcripts annotated with different databases. (B) Percentage of total TPM explained by the transcripts annotated with KEGG. **Fig. S5.** Overlap in functional KEGG annotations in three groups of peptidases (cysteine, aspartyl and serine peptidases) using the whole metatranscriptomic dataset. Values represent the percentage of overlap for each KO pair computed as shared transcripts (i.e., transcripts annotated with both KOs) divided by the total transcripts of the KO displayed in the y-axis. Abbreviations: c (cysteine); s (serine); cp (carboxypeptidase). **Fig. S6.** Nucleotide identity of transcripts associated to 51 represented species in the metatranscriptomes. We selected species having a median identity higher than 99% (green bars), while we discarded the rest (red bars). **Fig. S7.** Expression dynamics of the 25 species with genomic data found in the metatranscriptomes. Abundance values represent pseudocounts per million, obtained after correcting the abundance profiles by gene lengths and sequencing depth (see Methods for details). Note that some species appear in several incubations. **Fig. S8.** Per-sample summary of the mapping of the unassembled metatranscriptomic reads to the database EukProt+SAGs using DIAMOND blastx. Only alignments with >90% query coverage are considered. **Fig. S9.** Histogram of the CDS identity between the genes of MAST-4A and MAST-4B species available in EukProt v3. Nucleotide percentage identities were calculated using blastn. **Fig. S10.** Expression of selected genes in species with different trophic modes separated by the state of the incubations. Points represent the relative expression of the gene in a single species and sample. Values were computed by dividing the expression of the selected gene by the total expression for each species and sample. Values are separated by the trophic mode of the species they come from (Table 2).

**Additional file 2: Table S1.** List of the 359 most expressed genes (KO, KEGG Orthologs) in “growth” samples and general gene expression statistics associated.

**Additional file 3: Table S2.** Statistical test supporting the selection of candidate genes related to bacterivory. The two first columns show the number of gene expression observations for the 12 heterotrophic and 5 phototrophic species in all possible samples (dots in Fig. 5). For genes with at least 15 observations in the two groups, we performed a randomization test with 10000 permutations to test whether each gene was more expressed in heterotrophs than in phototrophs. In addition, when there was a much larger number of observations in heterotrophs than in phototrophs, the gene was considered to be more expressed in heterotrophs. In this selection, we also included the patterns of overexpression reported in Fig. 3 displayed in the ‘FC > FC(HK)’ column (‘Yes’ represents overexpression in at least 3 out of 4 incubations). The data leading to the negative selection form this list are marked in red.

## Data Availability

Raw data for “Jul17” incubation were already published at the NCBI BioSample database with accession number SAMN11783926 [38]. For the rest of the incubations, raw data are deposited at NCBI with accession number PRJNA973582. Assemblies, quantification, and functional annotation tables are available at FigShare (10.6084/m9.figshare.22801697), and all code used for data processing and analyses is available at GitHub (https://github.com/aleixop/metaT_bacterivory).
