# Supplementary material for: Gene expression dynamics of natural assemblages of heterotrophic flagellates during bacterivory
Source: Microbiome. 2023 Jun 15;11:134. doi: 10.1186/s40168-023-01571-5 (PMC10268365; doi:10.1186/s40168-023-01571-5)
Supplement: Supplementary file 2 — Additional file 1: Fig. S1. Overview of the experimental setup. Fig. S2. Overview of the bioinformatic processing of the metatranscriptomic reads. Fig. S3. PCA plots used for validation of the different incubation states based on normalized read counts per transcript. Each point represents a sample, and the value next to it represents the time of incubation expressed as days. Fig. S4. (A) Percentage of transcripts annotated with different databases. (B) Percentage of total TPM explained by the transcripts annotated with KEGG. Fig. S5. Overlap in functional KEGG annotations in three groups of peptidases (cysteine, aspartyl and serine peptidases) using the whole metatranscriptomic dataset. Values represent the percentage of overlap for each KO pair computed as shared transcripts (i.e., transcripts annotated with both KOs) divided by the total transcripts of the KO displayed in the y-axis. Abbreviations: c (cysteine); s (serine); cp (carboxypeptidase). Fig. S6. Nucleotide identity of transcripts associated to 51 represented species in the metatranscriptomes. We selected species having a median identity higher than 99% (green bars), while we discarded the rest (red bars). Fig. S7. Expression dynamics of the 25 species with genomic data found in the metatranscriptomes. Abundance values represent pseudocounts per million, obtained after correcting the abundance profiles by gene lengths and sequencing depth (see Methods for details). Note that some species appear in several incubations. Fig. S8. Per-sample summary of the mapping of the unassembled metatranscriptomic reads to the database EukProt+SAGs using DIAMOND blastx. Only alignments with >90% query coverage are considered. Fig. S9. Histogram of the CDS identity between the genes of MAST-4A and MAST-4B species available in EukProt v3. Nucleotide percentage identities were calculated using blastn. Fig. S10. Expression of selected genes in species with different trophic modes separated by the state of the incubations. Po [file 40168_2023_1571_MOESM1_ESM.pdf]

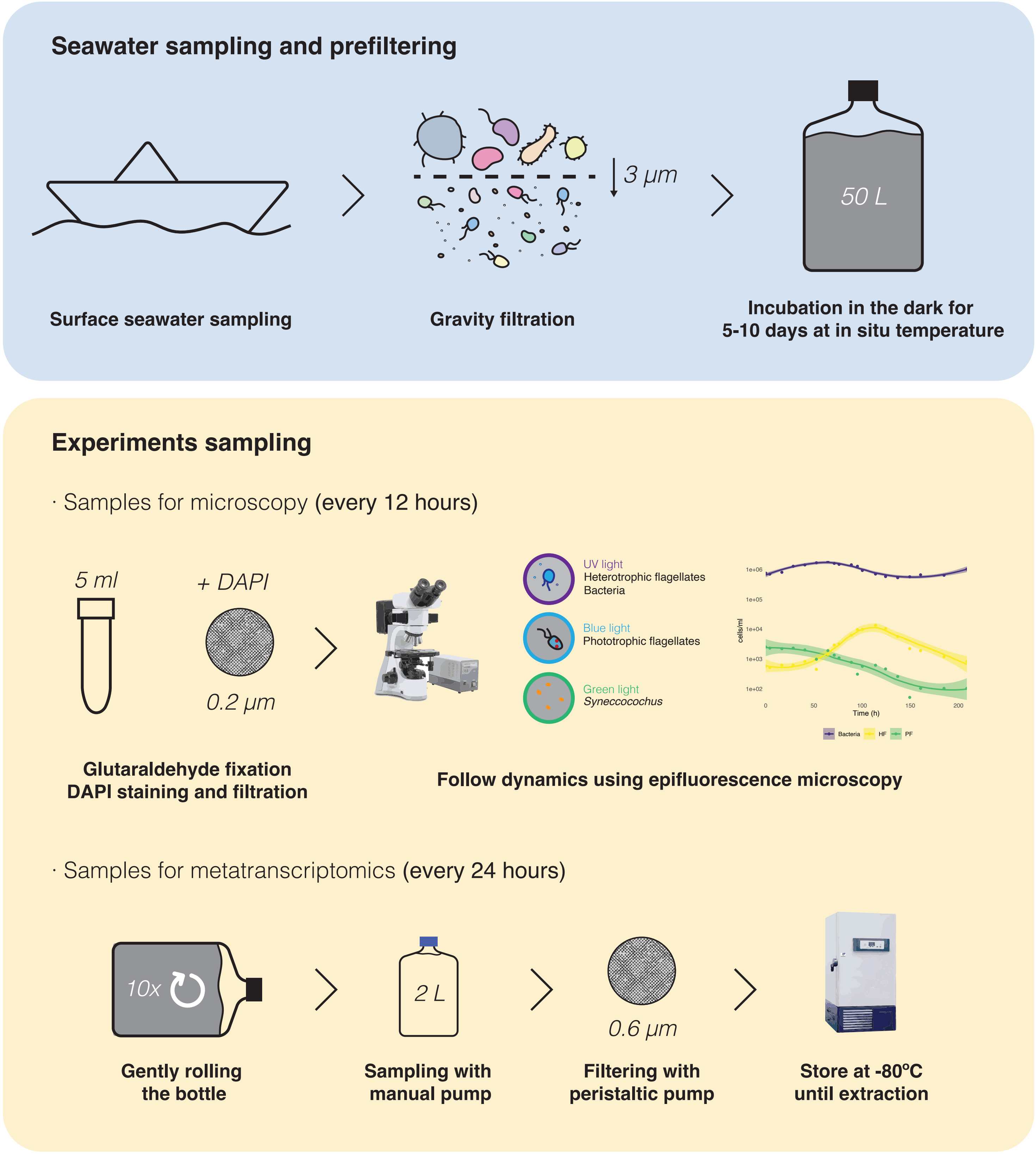
Seawater sampling and prefiltering
3 µm
50 L
Surface seawater sampling
Gravity filtration
Incubation in the dark for
5-10 days at in situ temperature
Experiments sampling
· Samples for microscopy (every 12 hours)
5 ml
+ DAPI
0.2 µm
UV light
Heterotrophic flagellates
Bacteria
Blue light
Phototrophic flagellates
Green light
Syneccocochus
1e+06
1e+05
1e+04
1e+03
1e+02
cells/ml
0
50
100
150
200
Time (h)
Bacteria
HF
PF
Glutaraldehyde fixation
DAPI staining and filtration
Follow dynamics using epifluorescence microscopy
· Samples for metatranscriptomics (every 24 hours)
10x
2 L
0.6 µm
Gently rolling
the bottle
Sampling with
manual pump
Filtering with
peristaltic pump
Store at -80ºC
until extraction

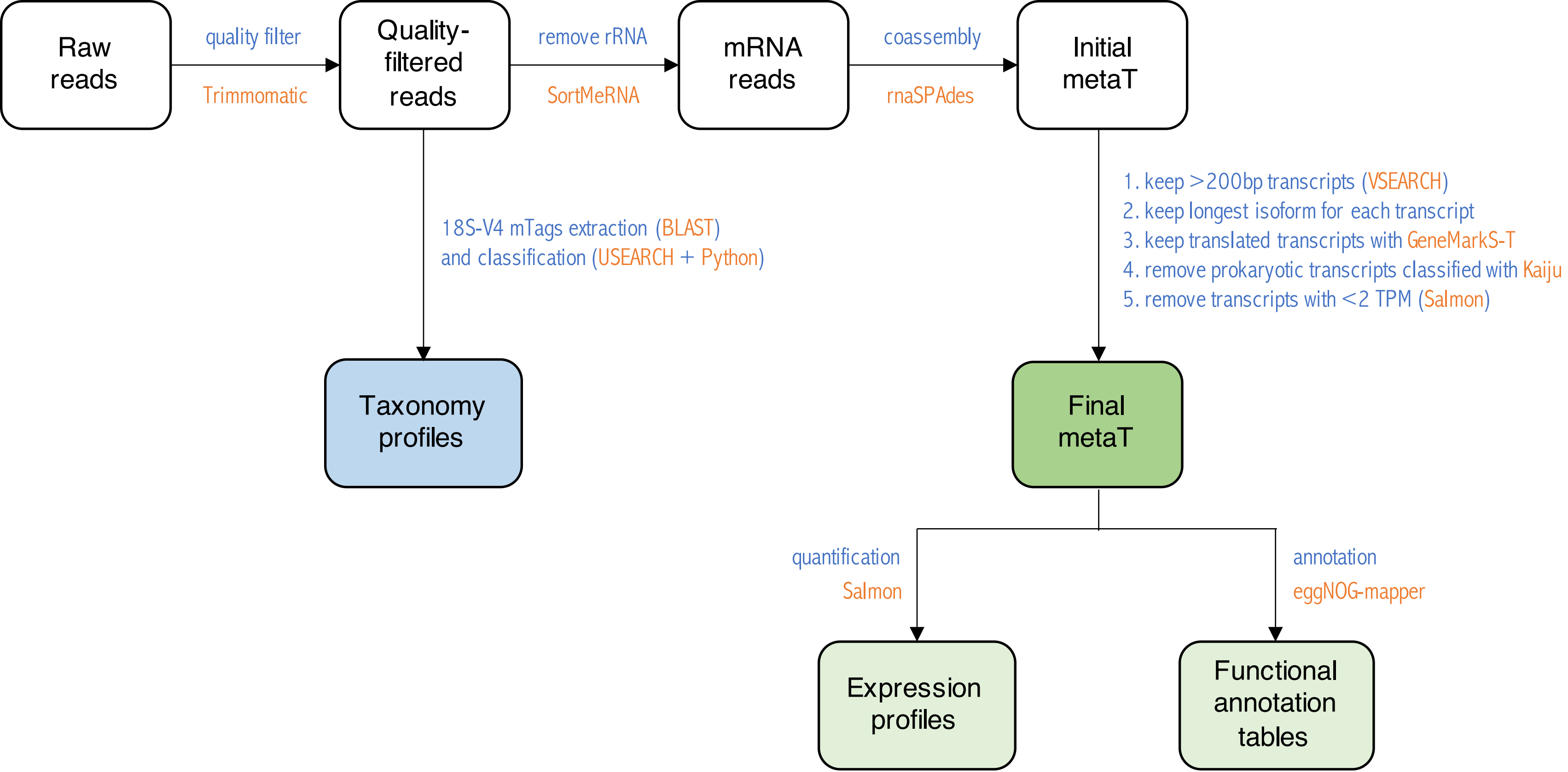
Raw reads
quality filter
Trimmomatic
Quality-filtered reads
remove rRNA
SortMeRNA
mRNA reads
coassembly
rnaSPAdes
Initial metaT
18S-V4 mTags extraction (BLAST)
and classification (USEARCH + Python)
1. keep >200bp transcripts (VSEARCH)
2. keep longest isoform for each transcript
3. keep translated transcripts with GeneMarkS-T
4. remove prokaryotic transcripts classified with Kaiju
5. remove transcripts with <2 TPM (Salmon)
Taxonomy profiles
Final metaT
quantification
Salmon
annotation
eggNOG-mapper
Expression profiles
Functional annotation tables

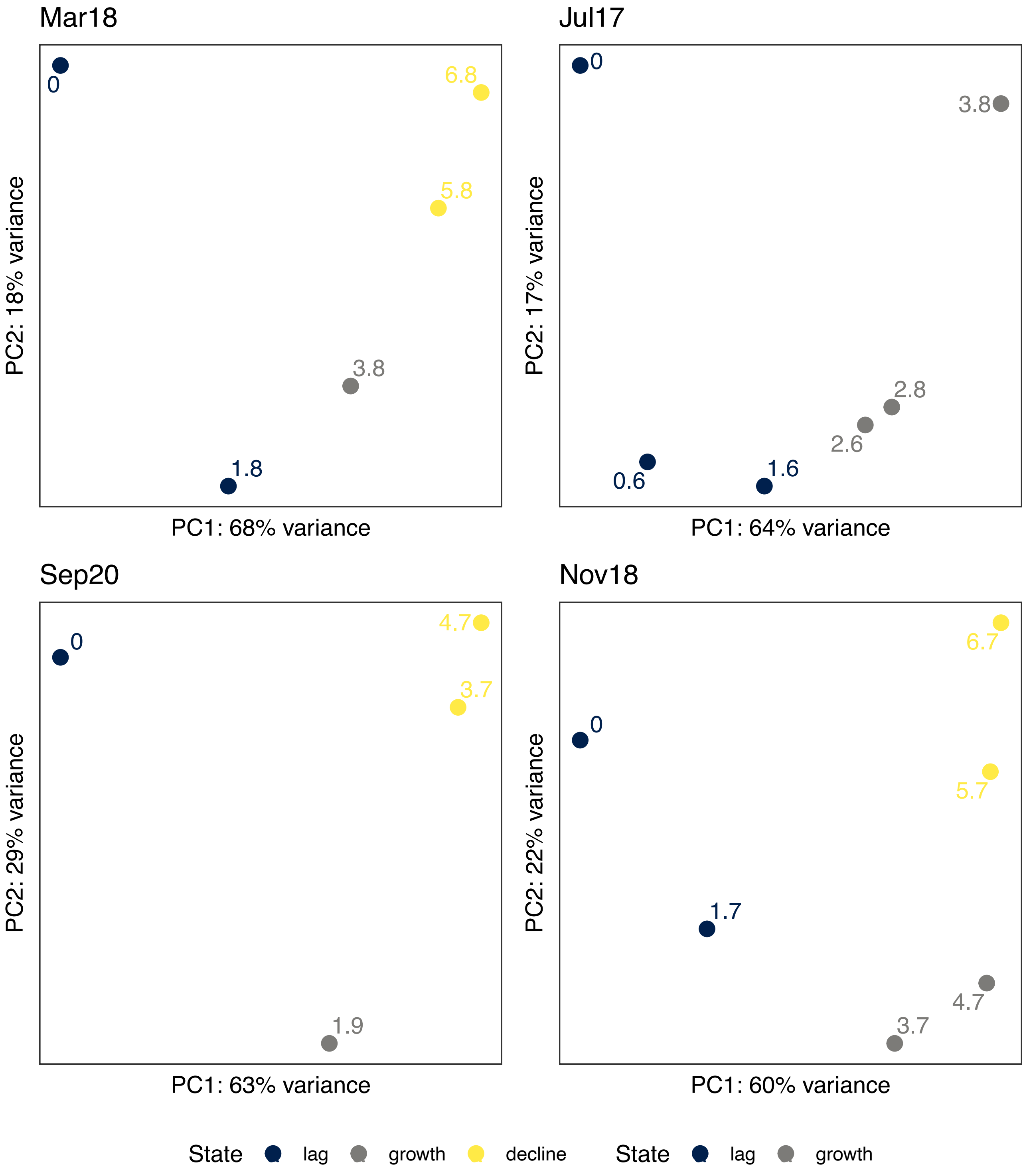

Mar18
0
6.8
5.8
3.8
1.8
PC2: 18% variance
PC1: 68% variance
Jul17
0
3.8
2.8
2.6
0.6
1.6
PC2: 17% variance
PC1: 64% variance
Sep20
0
4.7
3.7
1.9
PC2: 29% variance
PC1: 63% variance
Nov18
6.7
0
5.7
1.7
4.7
3.7
PC2: 22% variance
PC1: 60% variance
State lag growth decline
State lag growth

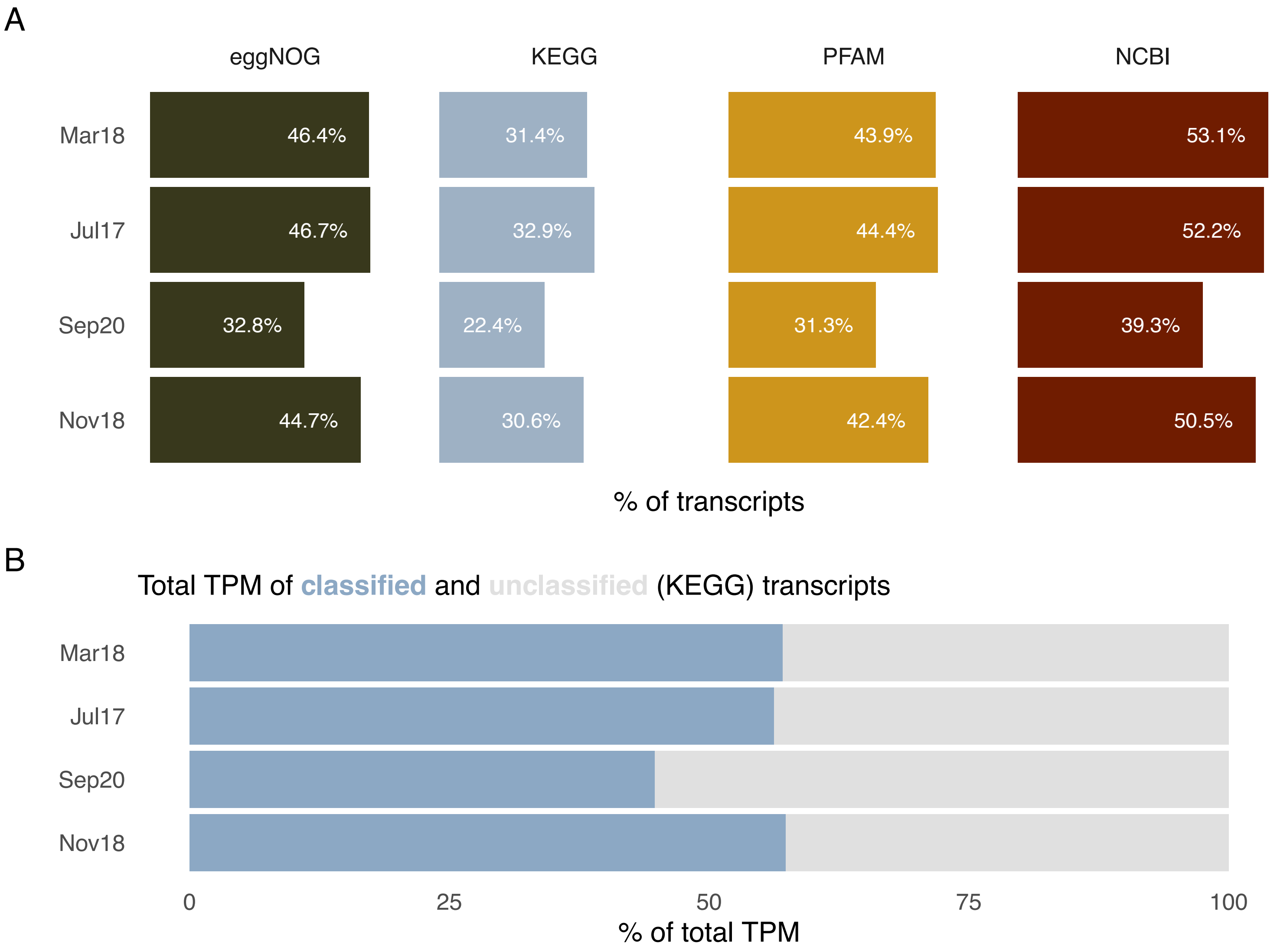
A
eggNOG
KEGG
PFAM
NCBI
Mar18
Jul17
Sep20
Nov18
46.4%
46.7%
32.8%
44.7%
31.4%
32.9%
22.4%
30.6%
43.9%
44.4%
31.3%
42.4%
53.1%
52.2%
39.3%
50.5%
% of transcripts
B
Total TPM of classified and unclassified (KEGG) transcripts
Mar18
Jul17
Sep20
Nov18
0
25
50
75
100
% of total TPM

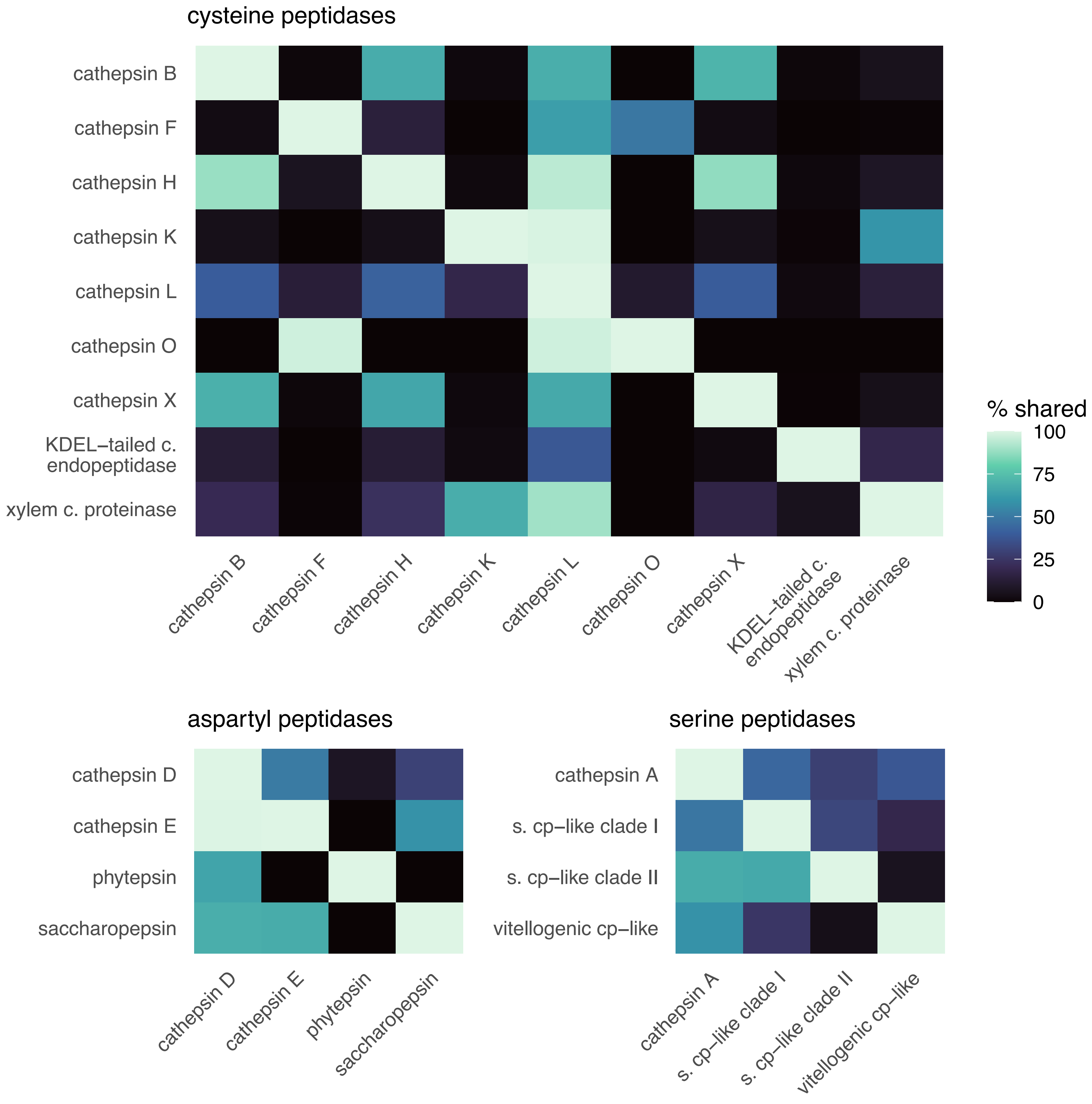
cysteine peptidases
cathepsin B
cathepsin F
cathepsin H
cathepsin K
cathepsin L
cathepsin O
cathepsin X
KDEL−tailed c. endopeptidase
xylem c. proteinase
% shared
100
75
50
25
0
aspartyl peptidases
cathepsin D
cathepsin E
phytepsin
saccharopepsin
serine peptidases
cathepsin A
s. cp−like clade I
s. cp−like clade II
vitellogenic cp−like

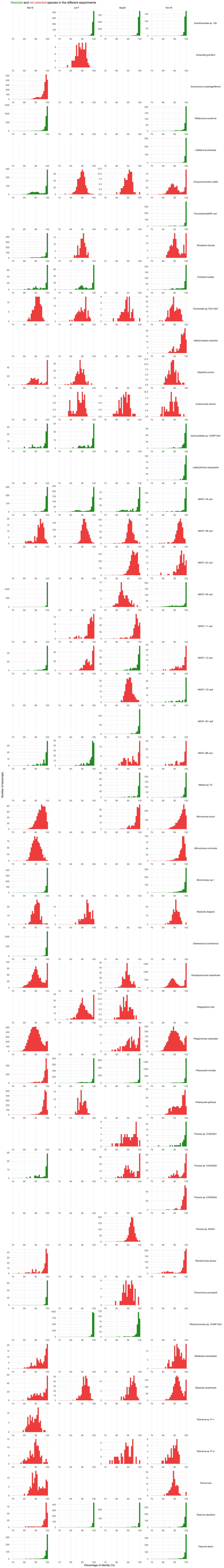

Selected and not selected species in the different experiments
Mar16
Jul17
Sep20
Nov18
Acanthoeca sp. 10tr
Amorella gravilens
Aureococcus anophagefferens
Bathycoccus prasinos
Calcidiscus leptoporus
Chrysochromulina rotalis
Chrysophyceae sp.
Dictyocha speculum
Emiliania huxleyi
Florenciella sp. RCC1007
Gephyrocapsa oceanica
Hapalosira ericina
Imantonia rotunda
Isochrysidales sp. CCMP1244
Leptocylindrus hargravesii
MAST-4A-sp1
MAST-4B-sp1
MAST-4C-sp1
MAST-4E-sp1
MAST-7A-sp1
MAST-1C-sp1
MAST-1D-sp2
MAST-3C-sp2
MAST-8B-sp1
Mataza sp. D1
Micromonas bravo
Micromonas commoda
Micromonas sp.1
Neobodo designis
Ostreococcus lucimarinus
Paraphysomonas imperforata
Pelagodinium beii
Pelagomonas calceolata
Phaeocystis cordata
Phaeocystis globosa
Picozoa sp. COSAG01
Picozoa sp. COSAG02
Picozoa sp. COSAG04
Picozoa sp. SAG01
Pteridomonas danica
Pyrenomonas provasoli
Rhizochromulina sp. CCMP1243
Skeletonema menzelii
Telonema antarcticum
Telonema sp. P-1
Telonema sp. P-2
Triparma laevis
Triparma eleuthera
Triparma laevis
Number of transcripts
Percentage of identity (%)

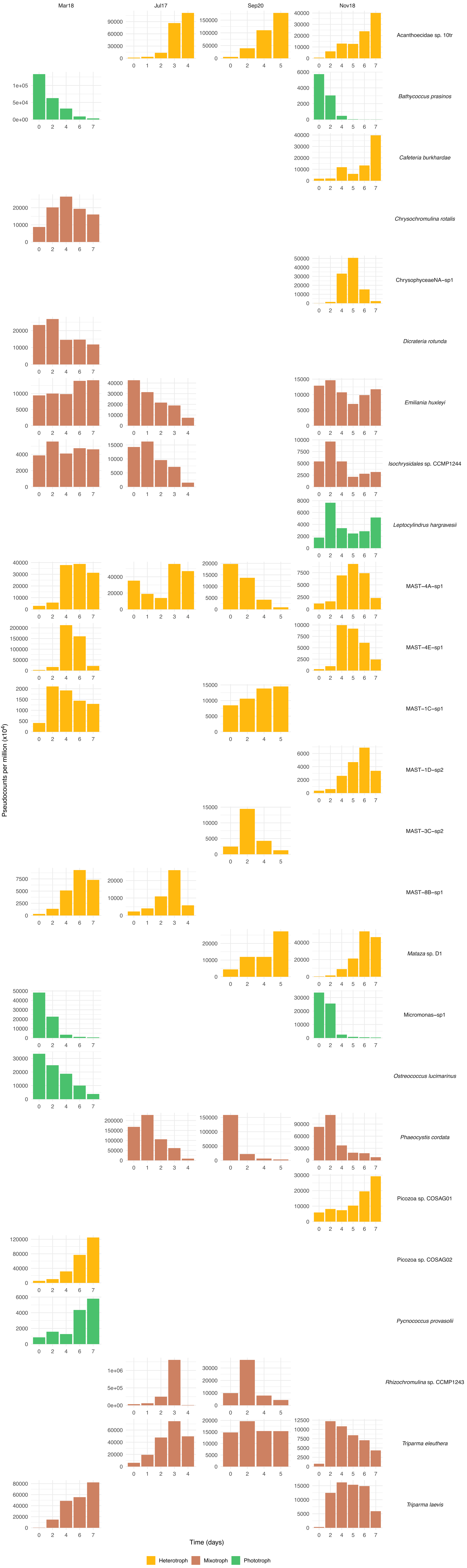
Mar18
Jul17
Sep20
Nov18
Acanthoecidae sp. 10tr
Bathycoccus prasinos
Cafeteria burkhardae
Chrysochromulina rotalis
ChrysophyceaeNA–sp1
Dicrateria rotunda
Emiliania huxleyi
Isochrysidales sp. CCMP1244
Leptocylindrus hargravesii
MAST–4A–sp1
MAST–4E–sp1
MAST–1C–sp1
MAST–1D–sp2
MAST–3C–sp2
MAST–8B–sp1
Mataza sp. D1
Micromonas–sp1
Ostreococcus lucimarinus
Phaeocystis cordata
Picozoa sp. COSAG01
Picozoa sp. COSAG02
Pycnococcus provasolii
Rhizochromulina sp. CCMP1243
Triparma eleuthera
Triparma laevis
Pseudocounts per million (x10^4)
Time (days)
Heterotroph
Mixotroph
Phototroph

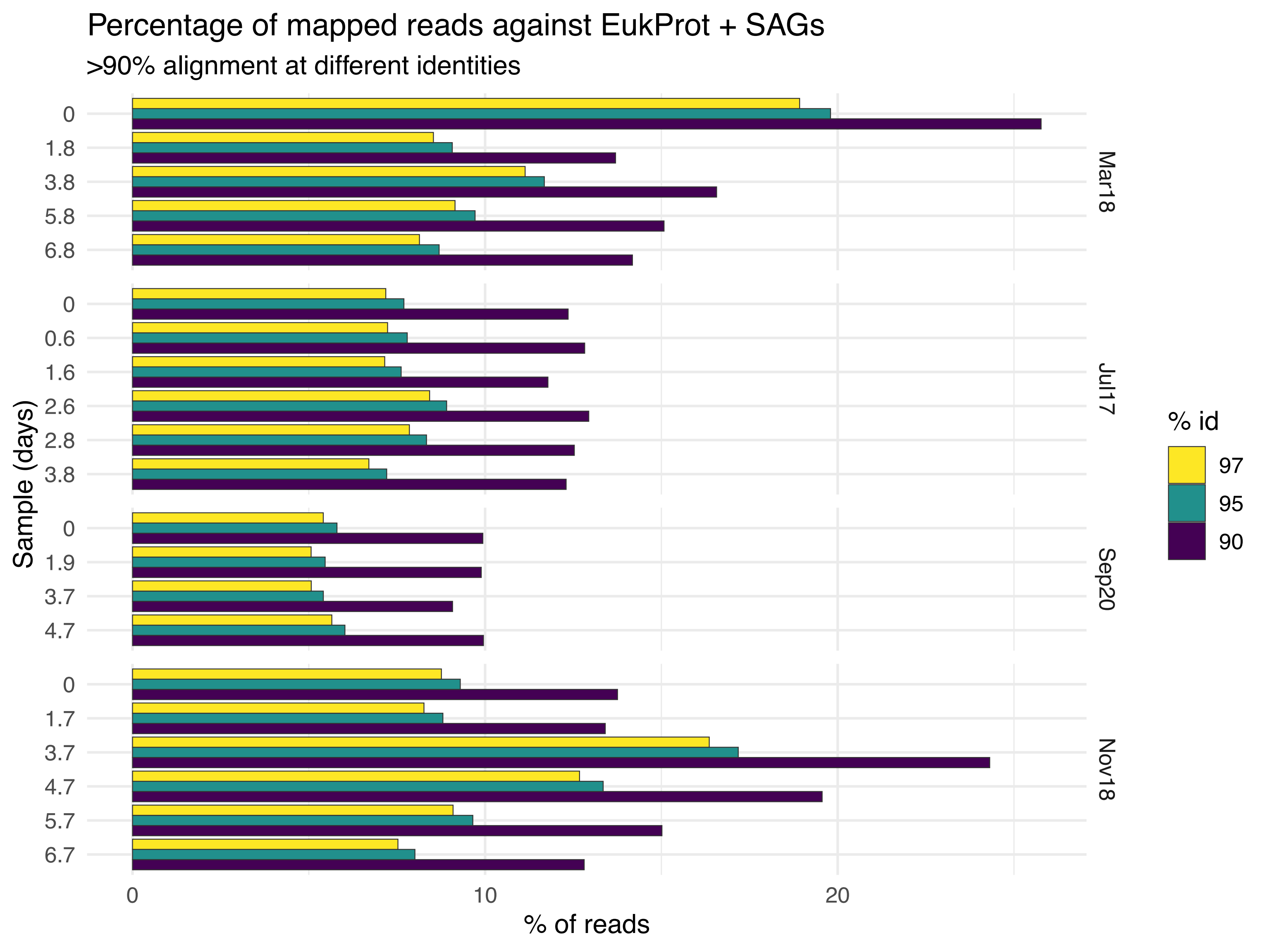

Percentage of mapped reads against EukProt + SAGs
>90% alignment at different identities
Sample (days)
0
1.8
3.8
5.8
6.8
Mar18
0
0.6
1.6
2.6
2.8
3.8
Jul17
0
1.9
3.7
4.7
Sep20
0
1.7
3.7
4.7
5.7
6.7
Nov18
0
10
20
% of reads
% id
97
95
90

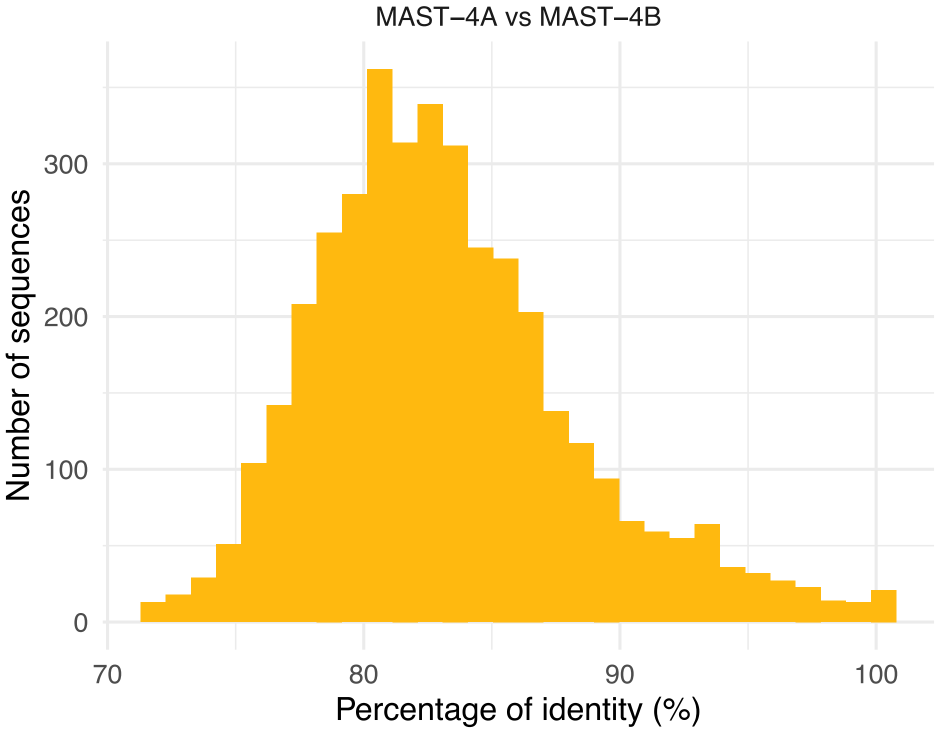

MAST−4A vs MAST−4B
Number of sequences
300
200
100
0
70
80
90
100
Percentage of identity (%)

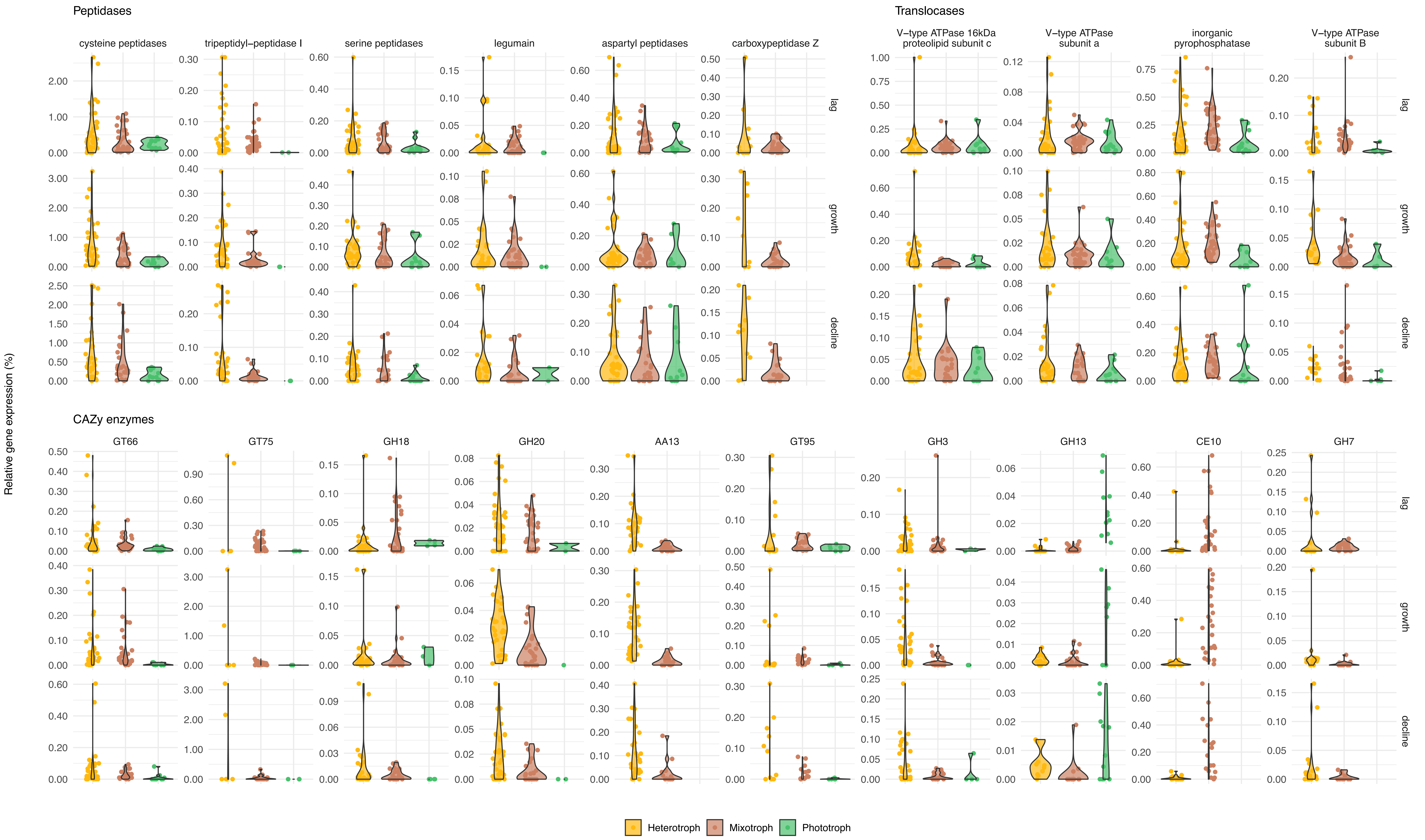

Peptidases
Translocases
CAZy enzymes
cysteine peptidases
tripeptidyl−peptidase I
serine peptidases
legumain
aspartyl peptidases
carboxypeptidase Z
V−type ATPase 16kDa proteolipid subunit c
V−type ATPase subunit a
inorganic pyrophosphatase
V−type ATPase subunit B
GT66
GT75
GH18
GH20
AA13
GT95
GH3
GH13
CE10
GH7
lag
growth
decline
Relative gene expression (%)
Heterotroph
Mixotroph
Phototroph
